# Small molecule-mediated regenerative engineering for craniofacial and dentoalveolar bone

**DOI:** 10.3389/fbioe.2022.1003936

**Published:** 2022-11-02

**Authors:** Juan Mitchell, Kevin W. H. Lo

**Affiliations:** ^1^ School of Dental Medicine, University of Connecticut Health Center, Farmington, CT, United States; ^2^ School of Medicine, Connecticut Convergence Institute for Translation in Regenerative Engineering, University of Connecticut Health Center, Farmington, CT, United States; ^3^ Department of Medicine, Division of Endocrinology, School of Medicine, University of Connecticut Health Center, Farmington, CT, United States; ^4^ Department of Biomedical Engineering, School of Engineering, University of Connecticut, Storrs, CT, United States; ^5^ School of Engineering, Institute of Materials Science (IMS), University of Connecticut, Storrs, CT, United States

**Keywords:** small molecules, regenerative engineering, drug discovery and delivery, craniofacial bone, dental materials, dentoalveolar

## Abstract

The comprehensive reconstruction of extensive craniofacial and dentoalveolar defects remains a major clinical challenge to this day, especially in complex medical cases involving cancer, cranioplasty, and traumatic injury. Currently, osteogenic small molecule-based compounds have been explored extensively to repair and regenerate bone tissue because of their unique advantages. Over the past few years, a number of small molecules with the potential of craniofacial and periodontal bone tissue regeneration have been reported in literature. In this review, we discuss current progress using small molecules to regulate cranial and periodontal bone regeneration. Future directions of craniofacial bone regenerative engineering using the small molecule-based compounds will be discussed as well.

## Background

The multi-faceted complex of craniofacial anatomy and dentoalveolar tissues (referring to the teeth and neighboring bone) contrives a plethora of intricate functions that are undeniably integral to the maintenance of systems throughout the body. The craniofacial bones composing the upper skull are known to provide immediate protection to the brain and other sections of the central nervous system while also shielding numerous sensory fibers responsible for major perceptive capabilities like olfaction, vision, auditory processing, and gustation. Craniofacial and dentoalveolar bones of the lower skull provide a rigid and fortuitous framework for soft tissues regularly used in the mastication and swallowing of food, production of speech, respiration, and formation of facial expressions (Anderson et al., 2021). Defects or lesions of these regions are often characterized by extensive trauma, malformation, or pathology affecting bony structures comprising the skull; as such, they are known to exist as especially debilitating hindrances that ascribe lasting functional, esthetic, and social barriers to patients who unwillingly incur a diminished quality of life because of them (Manlove et al., 2020). Examples of blatant and insidious impediments commonly braced by these patients include restricted communication, dysphagia, entropion, and ectropion, hindered temporomandibular joint mobility, reduced interpersonal relations, pronounced sentiments of ostracism, etc. Ubiquitous across many global populations, craniofacial and dentoalveolar anomalies may be observed in child and adult demographics alike—some such anomalies include congenital and idiopathic forms of orofacial clefting which affects 1 in every 700 live births, Treacher-Collins syndrome that leads to underdevelopment of facial bones and tissues responsible for proper maturation of the zygomatic bone and jawbones, Apert syndrome that yields an underdeveloped maxilla and misalignment of the teeth on account of premature suture closure, among several other oppressive disorders and acute traumatic injuries that result in a distinct loss of bone structure. In many cases, natural processes of bone healing are sufficient in restoring smaller lesions. Specifically, incumbent mesenchymal stem cells can undergo differentiation into regulatory osteoclasts and osteoblasts imperative for bone remodeling, complimented by increased activity of numerous cytokine-releasing lymphoid cells and growth factors that promote bone formation. While these types of subtle, confined, or localized defects are typically amenable through intrinsic pathways of biological bone repair, particularly large craniofacial and dentoalveolar defects often exhibit insufficiency of innate regeneration as the extent of involvement exceeds the body’s natural capabilities of osteogenic repair. With this in mind, translational applications of tissue rehabilitation have been established in the form of a limited number of clinical interventions that have provided defect patients with more viable solutions for meeting their needs. Despite this, many patients burdened with conditions of large craniofacial and dentoalveolar defects involving lesion-prone structures like the calvaria, mandible, temporomandibular joint, palate, cranial sensory organs, cranial muscles, periodontium, or dentition of the skull are still confronted with enduring challenges regarding available treatment options and their associated drawbacks.

A few prominent modalities of treatment currently being used to address large craniofacial and dentoalveolar defects include reconstructive procedures based in the use of techniques like prosthetic obturation which invokes upon the integrative and ostensibly seamless attributes of compatible biomaterials in the reformation of lesions, as well as in the resolution of many functional impairments displayed by patients (e.g., 3D-printing technologies for fabrication of tissue-specific prostheses). However, methods of prosthetic obturation remain an imperfect solution with their own constraints. For example, patients with material-based prosthetic obturators must often have their prostheses removed for regular maintenance and cleaning. Such prostheses also do not change color in response to UV exposure or aging as would be apparent in adjacent tissues of the patient’s skin. Conversely, reconstructive practices embedded in principles of tissue obturation and grafting (e.g., tissue transfer, allogeneic grafting, and autogenetic grafting) are more aptly used in the correction of sizeable anatomical deformities without requisites for regular maintenance. They also appeal to concerns of functional esthetics by avoiding jarring discrepancies in coloring that juxtapose the appearance of surrounding tissues. In fact, the current gold standard of treatment for segmental craniofacial and dentoalveolar defects utilizes methods of tissue obturation, particularly autologous bone grafting techniques, oriented around the replacement of endogenous tissues and the preservation of functional capacity. It’s estimated that over two million bone grafting procedures are carried out annually across the globe, approximately 500,000 of those procedures occurring within the United Stated. Touting high success rates of site infusion, along with a list of profound biomechanical, osteoconductive, osteoinductive, and osteogenetic properties, autologous bone grafts have proven to be advantageous in more ways than one for effective reconstruction. The benefits afforded by autologous grafts have made them a favorable mode of treatment for both surgeons and patients, especially when considering the adaptive curtailments of more synthetic bone substitutes. Though still, even these grafting methods are not without their own caveats. Auspicious as this technique may seem, autologous bone grafts are continuously associated with various restrictions that bring a degree of ambiguity to their unadulterated incorporation into a patient’s treatment plan; for instance, these methods may suffer from a possible lack of adequate bone structure for graft sampling, increased donor site morbidity related to bone sample procurement, patient pain or discomfort, and even progressive bone resorption in some cases (Schmidt et al., 2020). Minor tangential complications such as impaired wound healing, temporary sensory loss, prolonged wound drainage, and superficial infection have also been reported post-op by patients treated with autologous bone grafts. The osteodinductive growth factor, bone morphogenetic protein-2 (BMP-2), as another source of treatment has been applied clinically as a substitute for bone grafts in procedures amending sumptuous bone lesions. In fact, evidence from previous clinical trials has shown that BMP-2 use decreases total operatory time and is even more effective in treating certain lesions than autologous grafting materials (James et al., 2016). The likelihood of secondary, post-op interventions also sees a considerable drop with a conversion from grafting to BMP-2. However, the clinical profile of BMP-2 is not devoid of its own blemishes. Potentially life-threatening complications associated with BMP-2 are commonly observed, effecting as high as 20%–70% of cases; side effects include ectopic bone formation, osteolysis and subsidence, unregulated inflammation, bone cyst formation, adipogenesis, etc., which have all been reported in patients treated with BMP-2. There also remains the concern of high structural integrity requirements for adjacent tissues and the implementation costs tied to clinical BMP-2 use.

In order to nullify some of the indelible pitfalls coupled with techniques of modern obturation and BMP-2, novel approaches centered around the use of regenerative engineering are being evaluated as less constrained alternatives, or supplements, to treatment. Fervent research into potent stem cell populations, biomimetic scaffolding, and more comprehensive interventions for large-scale bone regeneration, has helped us realize new innovative means for optimizing the care of patients presenting with such substantial and complex defects (Fan et al., 2017a; Shi et al., 2019; Zhang et al., 2020; Gregory et al., 2022; Shah et al., 2022; Zhang et al., 2022; Zhu et al., 2022). Namely, within the recent decade, advancements in the clinical aptitude of osteogenic small molecules and the delivery systems used to facilitate molecular targeting of specialized tissues have substantially bolstered prospects for new therapeutic treatments (Lo et al., 2012; Carbone et al., 2014; Laurencin et al., 2014; Lo et al., 2014; O’Neill et al., 2019; Lo, 2022).

**FIGURE 1:**
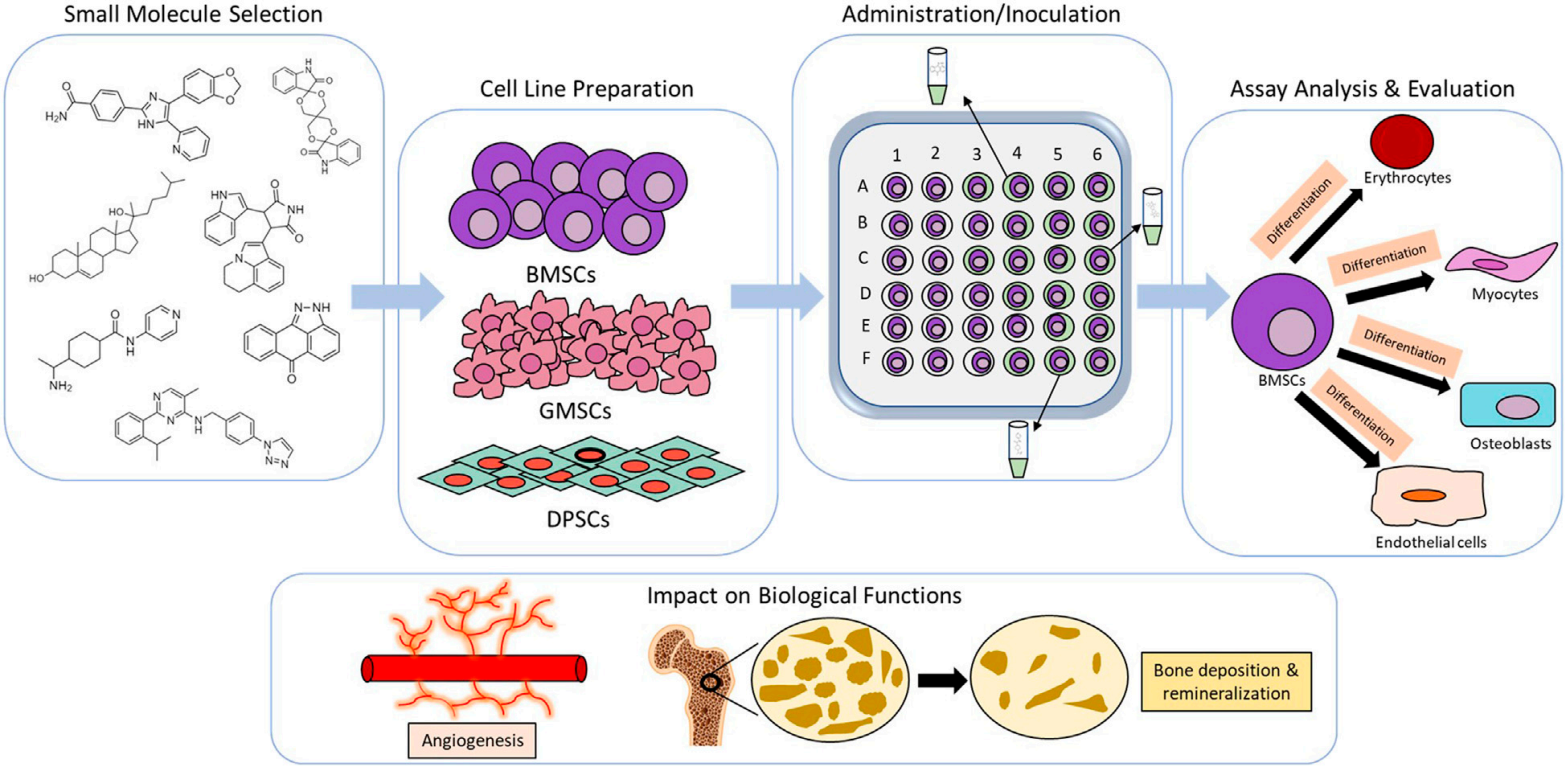
Example methodology of *in vitro* analysis for craniofacial and dentoalveolar tissue engineering. Meticulous selection of small molecules with favorable osteogenic properties and stem cell lineages from craniofacial or dentoalveolar tissues. Media imbued with small molecules are typically inoulated with stem cells and later assessed for expression of osteogenic markers and cellular differentiation. Cumulative change in biological functions (e.g.,angiogenesis and bone mineralization) are then evaluated.

**FIGURE 2:**
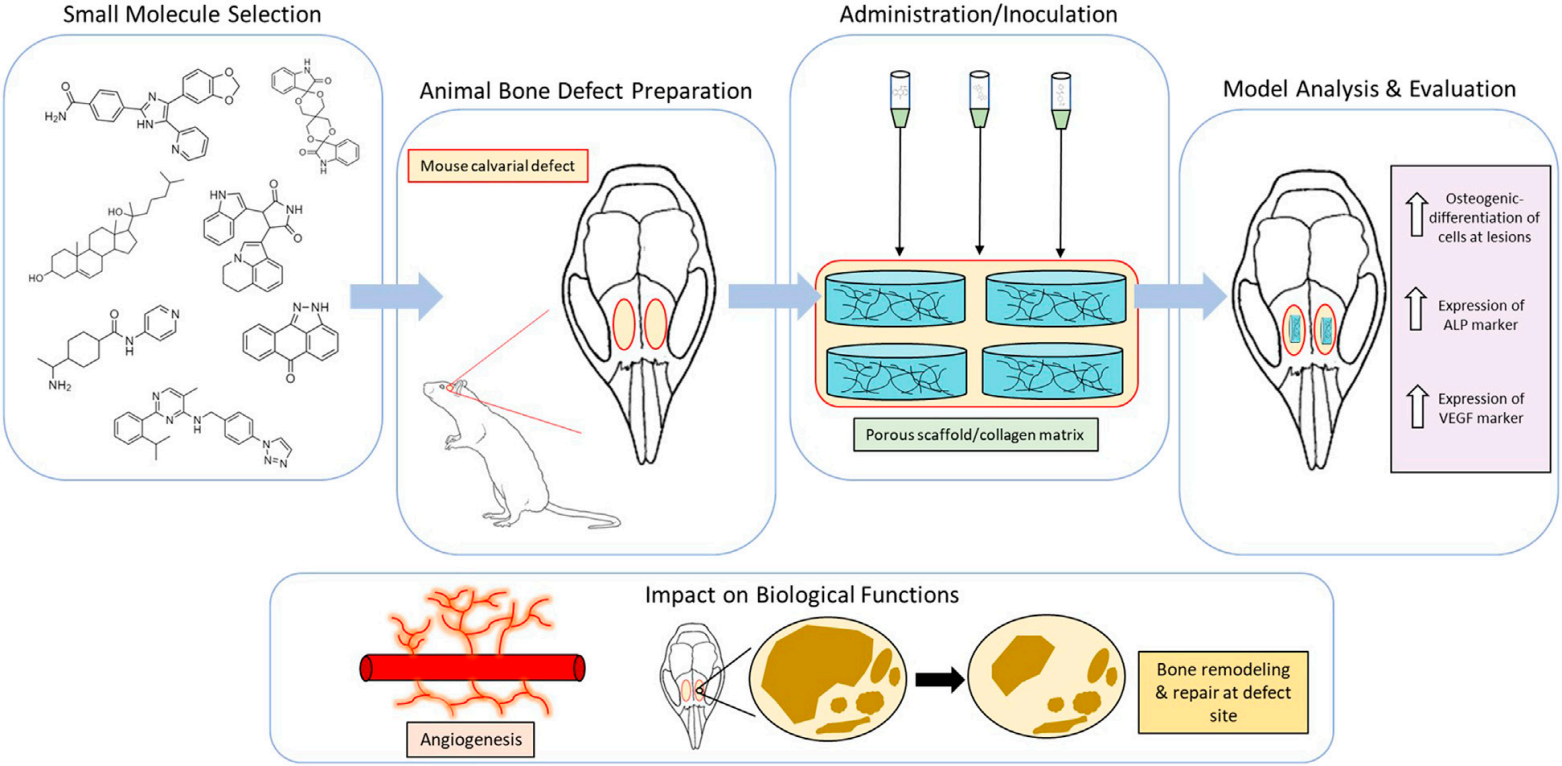
Example methodology of *in vivo* analysis for craniofacial and dentoalveolar tissue engineering. Meticulous selection of small molecules with favorable osteogenic properties and animal models involving defects of the craniofacial or dentoalveolar tissues. Scaffolds incorporated with small molecules are typically implanted at defect sites and later measured for expression of osteogenic markers. Cumulative change in biological functions (e.g.,angiogenesis and bone remodeling) are then evaluated.

Small molecules are defined as organic compounds of low molecular weight, expressly ≤1,000 Da, which include certain metabolites, drugs, monosaccharides, secondary messengers, and xenobiotics. Small molecules have largely seen use as favorable starting points in the discovery and development of new therapeutics for many chronic and acute conditions. Additionally, they have been utilized by some researchers as resourceful probes into the intricacies of less understood biological pathways. The regenerative properties demonstrated by some small molecules have shown promise in retaining the functional and physical traits of natural tissues observed at regions of craniofacial and dentoalveolar trauma or congenital defect while concomitantly ameliorating the risk of secondary infections, chronic pain, and other adverse effects that muddle the current index of treatments (Birjandi et al., 2020; Zhang et al., 2020; Shang et al., 2021). With the intent of enhancing osteoblast proliferation and effectively regulating osteoclast activity, a growing number of studies assessing the use of small molecules on large defects of the mandibular, maxillofacial, periodontal, and dental tissues have succeeded in prompting the osteogenic repair through various models. As a form of intervention, small molecules have also been shown to supplement angiogenesis and the differentiation of mesenchymal stem cells into osteoprogenitor cells (Segar et al., 2013; Ottensmeyer et al., 2018). At this time, there are many small molecule drugs that have already been awarded FDA approval following subjection to numerous trials for safety and efficacy assurance (De la Torre and Albericio, 2020). Notably, a vast majority of these small molecular agents also incur relatively low preparation costs, are easier to control, and can be conveniently synthesized in a lab or sourced from natural origins like plants and fruits (Awale et al., 2017). Through extensive *in vitro* and *in vivo* surveying of multiple biofactors and miniscule healing agents, the utility of small molecules in the regeneration of craniofacial and dentoalveolar tissues has become abundantly apparent and helped spur newfound hope for a better standard of care (Rodríguez-Méndez et al., 2018; Aghali, 2021; Emara and Shah, 2021) (Figures 1, 2). However, it must be noted that even though bioactive small molecules may potentially offer clinicians and their patients an array of propitious advantages, there still arises the question of proper dosing and delivery systems—specifically in regard to short-term and long-term drug administration (Song and Leeuwenburgh, 2014; Sarigol-Calamak and Hascicek, 2018; Liu F et al., 2021; Lo, 2022; Murugan et al., 2022). While these limitations require their own attention, the gradual expansion of small molecule therapeutics has still acted to further elucidate a multitude of burgeoning avenues through which clinically applicable tissue regeneration may be achieved in the near future for significant craniofacial and dentoalveolar defects (Ding et al., 2017; Mozaffari et al., 2019; Terauchi et al., 2021; Yi et al., 2020). Here, we discuss some of the recent strides made in using small molecules as a mode of rehabilitation for craniofacial and dentoalveolar tissues.

## *In vitro* evidence of small molecules on craniofacial and dentoalveolar bone regeneration

Techniques for small molecule screening such as cell panel screening are widely used for investigating *in vitro* biological mechanism and drug discovery (Figure 1). A number of groups have investigated the role of various glycogen synthase kinase-3 (GSK3)-inhibiting small molecules in the regeneration and differentiation of odontogenic stem cells, primarily endogenous to tissues of the dental pulp and dentin (Neves et al., 2017; Neves & Sharpe, 2018; Birjandi et al., 2020; Masuda et al., 2020). GSK3 has been identified as a key enzyme in the modulation of many signaling pathways responsible for the growth and maturation of bone, including those common to the formation of craniofacial and dentoalveolar tissues e.g., the Wnt/β-catenin pathway which has been demarcated as a pertinent cascade in stem cell differentiation and the regulation of dentin repair. One such study has shown that five unique small molecule drugs known to work through different mechanisms of action (Famotidine, Olanzapine, Naproxen, Cromolyn, and Tivantinib) are capable of acting as inhibitory agents against GSK3 (Birjandi et al., 2020). Of these drugs, Tivantinib—a non-ATP competitive small molecule mainly used in the treatment of hepatocellular carcinoma—was observed to incite *Axin2* expression and Wnt/β-catenin pathway signaling at a low concentration (50 nM) while simultaneously maintaining dental pulp cell viability through inconsequential levels of toxicity, thus potentially making it a suitable candidate for *in vivo* studies on the natural regeneration of odontoblasts necessary for reversing profound dentinal lesions and enhancing dentin preservation. In Masuda et al., *in vitro* cell culture treatments using the small molecule Tideglusib, another GSK3 antagonist, were seen to yield odontoblast-like cells with alkaline phosphatase (ALP)-positive calcified nodules in rat dental pulp cells after periods of intermittent and continuous exposure (Masuda et al., 2020). At a concentration of 50 nM, Tiderglusib significantly increased the number of calcified nodules observed among cultured pulp cells of the intermittent 6-h experimental group when compared with the control group. Though interestingly, a decline in the number of calcified nodules was reported among pulp cells of the continuous 48-h experimental group in comparison to the 6-h experimental and control groups. Additionally, the 6-h experimental group exhibited greater expression of the odontogenic biomarkers dentin sialophosphoprotein (Dspp) and osteocalcin (OCN) mRNA, while also promoting β-catenin aggregation and less β-catenin phosphorylation (i.e., minimal suppressor activity) within the cytoplasm of cultured pulp cells. The 48-h experimental group displayed less Dspp and OCN mRNA expression, along with more β-catenin phosphorylation. Purmorphamine, another osteoinductive small molecule with some conflicting findings across studies, has been shown to incite expression of osteoblastic phenotype markers in neural crest-derived dental pulp stem cells (DPSCs) (Rezia Rad et al., 2016). Elevated levels of DNA were examined among stem cells cultured on β-TCP granules with 2 µM Purmorphamine compared to cells cultured without 2 µM Purmorphamine, suggesting it plays a promotional role in the proliferation of DPSCs. In addition to promoting adhesion, proliferation, and differentiation of DPSCs cultured on β-TCP granules, Purmorphamine has been seen to increase the prominence of ALP, osteopontin (*OPN*), and *OCN* at both 7 and 14 days of induction. The small molecule ML323, a *USP1* inhibitor that prompts ubiquitination and degradation of the anti-osteogenic gene *ID1*, was also found to encourage osteogenesis of DPSCs during osteodifferentiation. ML323 increased ALP activity on day 14 of DPSC osteodifferentiation, complimented by calcium accumulation on day 21 (Kim and Choung, 2020). Similarly, the GSK3 inhibiting small molecule 6-Bromoindirubin-3′-Oxime (BIO) initiated osteodifferentiation of DPSCs in another study as evidenced by elevated ALP activity, mineral deposition, and osteogenic gene expression. BIO was seen to increase β-catenin translocation which upregulated the Wnt signaling pathway in the DPSCs. BIO also increased expression of osteogenic genes *ANKH, ENPP1, RUNX2, OSX, OCN, DSPP, DMP1,* and *OPG* paired with downregulation of osteoclastogenic RANKL (Kornsuthisopon et al., 2022).

It should also be noted that the stimulated differentiation of neural crest-derived DPSCs retains a unique advantage through the shared origin of DPSCs and other neural crest-derived craniofacial tissues, therein emboldening their candidacy in the development of regenerative therapies not limited solely to dental repair. The neurogenic aptitude of a specially tailored mosaic of small molecules was verified in Heng et al. (2019) through analyses on small molecule-induced differentiation of DPSCs, stem cells from apical papilla (SCAPs), and gingival mesenchymal stem cells (GMSCs) into neural cell lineages at 8 and 14 day checkpoints. A noteworthy increase in the presence of mature neural markers *NeuN*, *NFM*, *NSE*, and *MAP2* was observed in DPSCs and SCAPs, while other immature neural markers *Musashi1*, βIII-Tubulin, *NGN2*, and *MASH1* were seen in GMSCs. The small molecule cocktail used in this study (consisting of Valproic acid, Forskolin, SP600125, GO6983, Y-27632, Repsox, CHIR99021, Dorsomorphin) also showed ample apoptosis of initial stem cells consistent with the natural death of immature neural cells that precedes neuronal differentiation. Therapeutic applications hinging on the use of such neurogenic molecules suggest a feasible approach for the regeneration of nervous tissues incumbent to craniofacial and dentoalveolar bone. In another study by Shang et al. (2021), the prolyl hydroxylases (PHDs) inhibiting small molecule, Dimethyloxalylglycine (DMOG), paired with bioactive nanosilicate (nSi) in a polylactic acid/polyglycolic acid (PLGA) scaffold was also seen to be beneficial in the regeneration of periodontal tissues and remodeling of periodontal bone (Liu Z. Q et al., 2021). From the *in vitro* studies on this combination, an increase in ALP activity and other osteogenic markers like *Runx2*, *OPN*, *OCN*, and bone sialo-protein (BSP) were observed in the differentiation of periodontal ligament stem cells (PDLSCs) using DMOG/nSi scaffolds. With the expression of these markers, they noted an apparent formation of functional cementum-periodontal ligament alveolar bone complexes that further supported the occurrence of adequate differentiation. To complement these findings, sustained release of DMOG and profound degrees of angiogenic revascularization essential to periodontal healing were also apparent from these trials. Another integral approach to craniofacial bone regeneration and tooth repair explores the mechanisms of collagen biomineralization used in the preservation of innate functional and mechanical properties seen in many of the largely collagenous tissues of the cranium. Two other small molecules that have been shown to foster intrafibrillar mineralization include citrate and fluoride. The small molecule citrate, abundant across many craniofacial and dentoalveolar tissues, has been tokened as a purported osteopromotive factor that orchestrates the process of citrate metabolism for facilitating downstream effects on the differentiation of mesenchymal stem cells (MSCs) into osteoblasts and other osteogenic factors. In Ma et al., *in vitro* studies found that 200 µM citrate increased osteophenotype progression to amplify the expression of several osteogenic factors such as ALP, *OPN*, *Runx2*, *Col1a1*, and *SPP1* while also producing higher calcium/protein ratios (Ma et al., 2018). Other studies have also shown that citrate can prompt intrafibrillar mineralization by diminishing interfacial energy observed between collagen matrices and ACP precursors. Similarly, the inductive effects of fluoride in the synthesis and extrafibrillar deposition of collagen fibrils are seen to enhance extents of crystallization and rod-like mineralization (Saxena et al., 2018). Fluoride has already been evidenced to promote the remineralization of hard dental tissues (namely enamel and dentin with large compositions of inorganic material) through fluorapatite formation. Though it should be mentioned, that while fluoride does display components of collagen mineralization, it has largely been cited in dental cavity prophylaxis and dental repair only due to its lower solubility threshold and heightened hardness in relation to hydroxyapatite. Drug delivery techniques in craniofacial and dentoalveolar engineering have also been tested using small molecules. For instance, small molecule-incorporated scaffolds have been assessed for their use in the controlled release of agents intended to accelerate the proliferation and osteodifferentiation of stem cells. In Song et al. (2021), a small molecule TGF-β inhibitor, SB431542, was consorted with an injectable calcium phosphate cement (CPC) scaffold for *in vitro* analyses. These scaffolds were then evaluated for their utility in promoting the proliferation, differentiation, and viability of human-induced pluripotent stem cells (hiPSCs). Of the scaffolds tested in this study, the CPC scaffolds with integrated 100 µM SB431542 showed the highest prevalence of viable hiPSC-MSCs as well as the greatest effects on osteogenic differentiation in comparison to the CPC control scaffolds without SB431542. All scaffolds, including those embedded with the small molecule, displayed continuous release over the 14-day trial period as required of a satisfactory delivery system. In the absence of scaffolding, SB431542 was also observed to encourage osteogenic differentiation in mesoderm-derived parietal bone osteoblasts (PObs) and dura mater (DM) cells through TGF-β inhibition. Heightened BMP signaling paired with increased *OCN* and *Runx2* expression at days 12 and day 18 in SB431542-treated cell cultures, were demonstrated by PObs and DM cells of the experimental group during differentiation and time course assays. A reduction in activity of the crucial apoptotic protease Caspase 3 was also observed. As a point of interest, it was noted that SB431542 unexpectedly upregulated *Smad6* in both of the surveyed cell lines instead of downregulating its expression—refuting the prospect of reduced *Smad6* being the principal mechanism behind increased BMP signaling seen during SM431542-mediated inhibition of TGF-β (Senarath-Yapa et al., 2016). In Zhang et al. (2020), potential methods of advanced craniofacial reconstruction and regeneration using multi-material 3D printing techniques were explored. They developed polycaprolactone/β-tricalcium phosphate (PCL/TCP) and hydrogel-based bioink composite scaffolds embedded with two osteogenic small molecules, resveratrol (RSV) and strontium ranelate (SrRn), seen to promote bone formation and reduce bone resorption. Through *in vitro* analysis, it was demonstrated that RSV and SrRn scaffolds were effective in increasing ALP activity through BMP-2, inciting osteoblastic differentiation, upregulating Wnt/β-catenin signaling, spurring promotion of mineralization in mouse MSCs, among a slew of other osteogenic properties. Scaffolded combination of RSV and SrRn was also shown to exhibit a synergistic coalition in promoting angiogenesis while concurrently inhibiting degenerative osteoclast activity. In characterizing the release of these two small molecules, an initial burst of release followed by more sustained release for SrRn but consistent sustained release for RSV over the 3-week release period. Primary outcomes for in vitro trials are outlined in Table 1.

**TABLE 1:** Small molecular agents examined *in vitro*.

Molecule	Classification	Molecular Weight	Working Concentrations	Cell lineage/Specimen	Delivery Methods	Primary Osteogenic Effect(s)	References
Tivantinib	3-alkylindoles	369.4 g/mol	50 nM, 200 nM	17IA4 mouse dental pulp cells	Pulp cell culture using standard culture medium treated with tivantinib	*Axin2* and Wnt/β-catenin pathway upregulation	Birjandi et al. (2020)
Tideglusib	Naphthalenes	334.4 g/mol	50 nM	Rat dental pulp cells	Pulp cell culture using α-MEM medium treated with tideglusib	Promote odontoblast-like cell differentiation; ALP-positive calcified nodules in isolated rat dental pulp cells	Masuda et al. (2020)
Purmorphamine	Purine	520.6 g/mol	2 μM	Human DPSCs from soft tissue of extracted third molar	β-TCP granules cell-scaffolds cultured in stem cell growth or osteogenic media	Increased ALP activity; *OPN* , *OCN* upregulation; stem cell proliferation	Rezia Rad et al. (2016)
Chemical cocktail*	Assortment (see footnote)	Assortment (see footnote)	Assortment (see footnote)	Human DPSCs from soft tissue of extracted third molars, donated human SCAPs, donated GMSCs	Stem cell culture using standard medium treated with chemical cocktail	*NeuN, NFM, NSE, MAP2* expression in DPSCs and SCAPs; *Musashi1, βIII-Tubulin, NGN2, MASH1* expression in GMSCs	Heng et al. (2019)
Dimethyloxalylglycine	Glycine derivative	175.1 g/mol	1% w/w of PLGA	Human PDLSCs from periodontal ligament of extracted premolars	Stem cells were inoculation using α-MEM growth medium treated with DMOG	Increased ALP activity; *Runx2* , *OPN, OCN, BSP* expression	Shang et al. (2021)
Citrate	Tricarboxylic acids	189.1 g/mol	200 μM	Human MSCs from bone marrow of healthy, non-diabetic adults	Stem cells treated with an osteogenic medium supplemented with pH-adjusted citrate	Increased ALP activity; *OPN, Runx2, Col1a1, SPP1* expression; higher calcium/protein ratios	Ma et al. (2018)
Fluoride	Halogen	19 g/mol	Different concentrations between 0 ppm and 200 ppm	Tendons extracted from tails of 12–18 week old Sprague Dawley rats	Rat tendons mineralized in 50 mM Tris buffer with 0, 0.5, 1, 2,	Enhanced crystallization and rod-like mineralization	Saxena et al. (2018)
SB431542	Benzamides	384.4 g/mol	100 μM	Human iPSC-MSCs yielded from sub-culture of bone marrow hiPSCs in MSC medium	Stem cell culture using standard medium, then osteogenic medium after cells attached to CPC scaffold	Promotes stem cell proliferation, differentiation, and viability	Song et al. (2021)
.	.	.	10 μM	Human PObs obtained from child calvarial bone graft patients and mouse PObs harvested from the skulls of mice; Mouse DM cells harvested from dural membrane of mice	Calvarial cell culture using osteogenic differentiation medium treated with SB431542	Increased BMP signaling; increased OCN and Runx2 expression; reduction in activity of Caspase 3; upregulated Smad6	Senarath-Yapa et al. (2016)
Resveratrol	Stilbenes	228.2 g/mol	1.0 mg/ml	Mouse MSCs isolated from bone marrow flushes of female FVB mice	Stem cells were inoculated in growth medium with RSV and RSV-SrRn scaffolds	Increased BMP2 signaling; osteoblastic differentiation; Wnt/β-cateninsignaling upregulation; mineralization in mouse MSCs; angiogenesis	Zhang et al. (2020)
Strontium ranelate	Tetracarboxylic acids	513.5 g/mol	1.0 mg/ml	Mouse MSCs isolated from bone marrow flushes of female FVB mice	Stem cells were inoculated in growth medium with SrRn and RSV-SrRn scaffolds	Increased ALP activity; Inhibited degenerative osteoclast activity	Zhang et al. (2020)
ML323	-	384.5 g/mol	10 μM	Human DPSCs from soft connective tissue of third molars	Stem cells were inoculated in osteogenic differentiation medium treated with ML323	Increased ALP activity; increased calcium accumulation; enhanced osteodifferentiation	Kim and Choung. (2020)
6-Bromoindirubin-3'-	Indole derivative	356.2 g/mol	200 nM, 400 nM, 800 nM	Human DPSCs from soft connective tissue of third molars Oxime	Stem cells were cultured using DMEM growth medium treated with BIO	Increased of ALP activity; ANKH, ENPP1, RUNX2, OSX, OCN, DSPP, DMP1, and OPG expression; RANKL downregulation; enhanced osteoblastic differentiation and mineralized nodule formation	Chatvadee et al. (2022)

*Valproic acid (Propylpentanoic acid derivative; 144.2 g/mol; 0.5 mM), Forskolin (Labdane diterpenoids; 410.5 g/mol; 10 μM), SP600125 (Anthrapyrazoles; 220.2 g/mol; 10 μM), GO6983 (Maleimides; 442.5 g/mol; 5 μM), Y-27632 (N-arylamides; 247.3 g/mol; 5 μM), Repsox (Pyrazolopyridine.; 287.3 g/mol; 1 μM), CHIR99021 (Aminopyrimidines; 465.3 g/mol; 3μM), Dorsomorphin (Pyrazolopyridine; 399.5 g/mol; 1 μM).

## *In vivo* evidence of osteogenic small molecules on craniofacial and dentoalveolar bone regeneration

**FIGURE 3:**
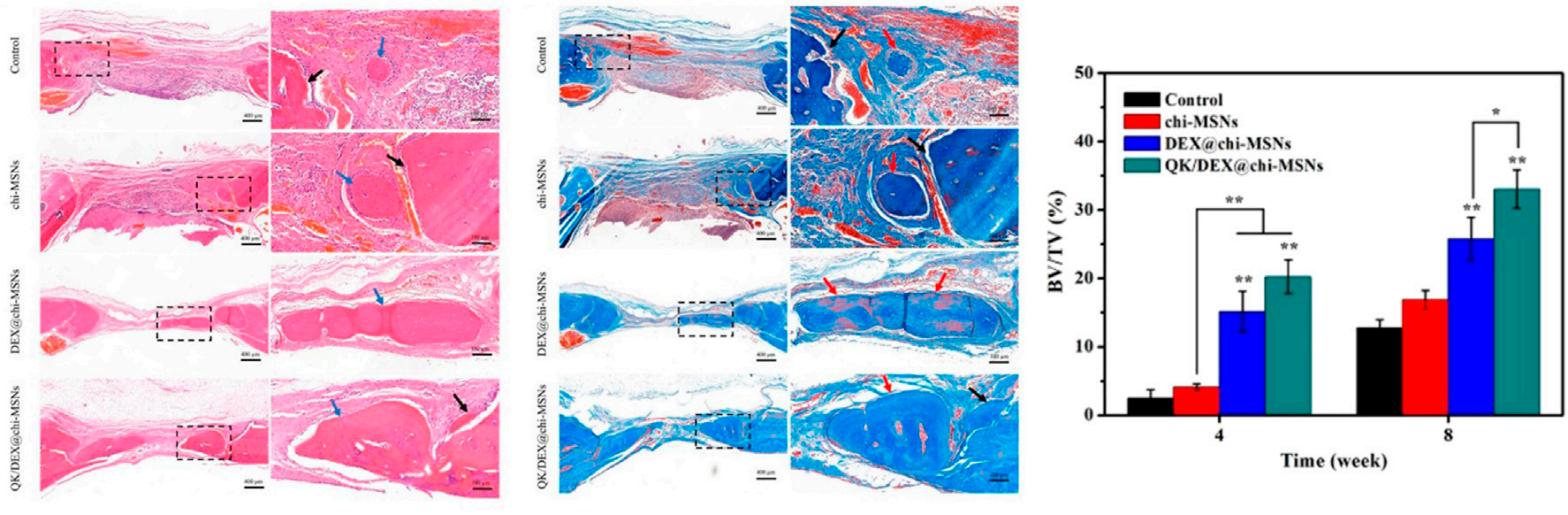
Comparative H&E and Masson’s trichrome stains characterizing new bone formation in rat cranial bone defects treated with small molecule dexamethasone (DEX)-incorporating gelfoams (DEX/@chi-MSNs; QK/DEX@chi-MSNs) in addition to DEX-devoid gelfoams (Control; Chi-MSNs), 8 weeks after placement within defect. Blue arrow represent new bone, while black arrows represent incumbent host bone. Using CT-Analyser Software, elevated bone volume/total volume ratios were measured in treatment groups incorporating DEX. Reproduced from (Sun et al., 2019)

In pursuit of more pragmatic modes of treatment and therapy for patients suffering from large craniofacial and dentoalveolar bone lesions, it is commonplace that inquiries into the assimilative aptitude of these small molecular agents be brought into question. Currently, a number of studies have proven that many of the osteogenic attributes provided by small molecular agents are steadfast in animal models seen to effectively mimic the microenvironment and stromal interactions seen in settings of significant bone loss and defect (Figure 2). One such study has assessed the osteogenic properties of SB431542 in minipigs with craniofacial bone lesions. Shi et al. (2019) postulates that SB431542 downregulates canonical TGF-β signaling by competitively binding to TGF-β type I receptors in order to prevent *Smad3* gene phosphorylation and induce activation of BMP signaling. They also found that this small TGF-β inhibitor acts as a catalyst in the differentiation of iPSCs to human gingival mesenchymal stem cells (hGMSCs) while also substantially amplifying BMP-2 and BMP-4 expression in immature osteoblasts for enhanced regeneration of large maxillofacial defects. In fact, *in vivo* and *in vitro* trials utilizing 1 µM SB431542 were observed to elicit a number of osteogenic effects; reduced hGMSC apoptosis rates, extensive osteogenic differentiation of hGMSCs, increased subcutaneous osteogenesis of hGMSCs in nude mice, and elevated bone regeneration of autologous GMSCs in the presence of defects. In other trials with SB431542, an increased rate of bone healing was established in mice with parietal bone defects that were a part of the experimental group as opposed to those of the control group. This was confirmed using immunohistochemistry analysis of the dural membrane extending these calvarial defects which showed a greater reduction in pSmad2/3 staining for the SB431542-treated group (Senarath-Yapa et al., 2016). In a study using rats with alveolar cleft defects branching from the zygomatic arch to the ipsilateral maxillary incisor, collagen sponges treated with a hydroxycholesterol mixture comprised of the small molecules 20(S)-hydroxycholesterol and 22(R)-hydroxycholesterol was the most effective in promoting osteogenic bone formation among all other treatment and control groups. Specifically, the hydroxycholesterol mixture exhibited the highest trabecular number and thickness in morphometric analysis while also retaining the lowest trabecular separation. The histological presence of pseudostratified columnar epithelium on the side of the nasal cavity in hydroxycholesterol-treated defect models indicated the lack of any adverse inflammatory reactions spurred by this mixture of small molecules (Bakshi et al., 2019). The small molecule phenamil, an amiloride derivative, is shown to prompt osteogenic differentiation of mesenchymal progenitors to encourage bone regeneration in animal models with large craniofacial defects. It is believed that phenamil accomplishes this through tribbles homolog 3 (Trib3)-incited inhibition of *Smad* ubiquitin regulatory factor 1 (Smurf1) that negatively regulates BMP-receptor related *Smad*s. In Fan et al., trials administered on large rat mandibular defects using 300 µM phenamil were seen to yield almost complete formation of trabeculae and bone volume at lesion sites, even when paired with low doses of BMP-2 (Fan et al., 2017b). Fascinatingly, doses of BMP-2 seen to produce hollow cysts containing fatty marrow and peroxisome proliferator activated receptor gamma (PPARγ)—both being markers of BMP-induced adipogenesis and inflammation—were not observed to produce such cysts when introduced with phenamil. Thus, phenamil was not only seen to promote BMP-induced osteogenesis but also circumvent some of the adverse manifestations of BMP-2 based therapies (i.e., adipogenesis and inflammation). Also, when delivered alongside noggin siRNA in sterosomes containing stearylamine and cholesterol, phenamil benefits from a synergistic increase in MSC osteogenesis and bone formation (Cui et al., 2017). In the aforementioned Zhang et al. (2020) study employing RSV-SrRn scaffolds, *in vivo* implantation trials on rat models with critical-sized mandibular defects showed increased bone formation following RSV-SrRn scaffold introduction. From baseline, comparatively higher bone volume to total volume (BV/TV) ratios were observed in scaffolds integrated with the RSV-SrRn combination as opposed to those control scaffolds without small molecule incorporation—therein supporting the *in vivo* efficacy of this small molecule combination. Also noted from their study, peripheral and central coverage of bone regeneration in large mandibular defects was evident using the RSV-SrRn dual small molecule scaffold. Dexamethasone (DEX), a small osteogenic hydroxysteroid, has also been shown to promote bone healing in rats with skull defects when delivered using chitosan-modified mesoporous silica nanoparticles (chi-MSNs). At 4 weeks, rat models treated with DEX@chi-MSNs demonstrated a significant increase in BV/TV ratios which was not mimicked in those models treated with the chi-MSN control vacant of DEX. After 8 weeks, elevated bone formation was observed in all groups, including the control; however, those incorporating DEX were still seen to entice the most growth (Sun et al., 2019) (Figure 3). In a similar manner, *in vivo* studies conducted on the ROCK (Rho-associated coiled-coil kinase) inhibitors HA-1077 (Fasudil) and Y-27632 showed that these small molecular agents were capable of accelerating the induction of bone healing in rats with parietal bone defects. Using atelocollagen sponges that were injected with PBS, 0.5 µM HA-1077, or 0.5 µM Y-27632 and implanted at defect sites, it was determined that HA-1077 and Y-27632 both promoted a high degree of osteoregeneration over the span of 4 weeks. The expression of VEGF-A mRNA, an important marker of the osteogenic protein VEGF that is imperative for proper bone defect repair and formation, was detected in osteoblast progenitor cells derived from injured rat bone marrow treated with 5 µM Y-27632 (Nakata et al., 2020).

Furthermore, viable applications in the regeneration of large orofacial defects, like those of the alveolar bone, have also been explored using the small molecule drug simvastatin (SV) which is generally indicated in the treatment of cholesterol-mediated cardiovascular diseases. Along with its cholesterol reducing properties, simvastatin has been established as a prominent osteogenic agent capable of inducing BMP-2 gene expression to potentiate bone regeneration while also displaying some anti-resorption elements to impede osteoclast proliferation. In Wu et al. (2008), PLGA scaffolds incorporated with SV were administered to sites of residual alveolar ridge resorption following tooth extraction in rat models. Over a release period of 12 weeks, SV/PLGA scaffolds were seen to be much more successful at stimulating bone formation than non-treated control PLGA scaffolds. Particularly, SV/PLGA scaffolds exhibited higher residual alveolar ridge heights and bone mineral densities at all reference points for release. These findings have provided *in vivo* evidence of simvastatin’s distinct osteogenic propensities that may work to accelerate healing and bone deposition in cases of large orofacial bone loss. DMOG/nSi scaffolds were also seen to promote revascularization and bone regeneration in rats with periodontal and mandibular defects through stimulation of PDLSC osteogenic differentiation and modulation of deleterious inflammatory responses during tissue repair (Liu F. et al., 2021; Shang et al., 2021). In studies conducted by Neves et al. (2017), Tideglusib has also been explored in the incitement of tertiary dentin formation in deeply lesioned *in vivo* mouse molars due to the drug’s promotion of Wnt/β-catenin pathways in odontoblastic pulp cells (e.g., DSPCs) for elevated deposition of dentin matrix (Neves & Sharpe, 2018). Preliminary trials showed that Tideglusib, along with the small molecule GSK3 antagonists CHIR99021 and BIO, elicited increased formation of reparative dentin in injured molars with pulp exposure at both 4 and 6 weeks. Reconstruction of dentin tissues using these molecules was also paired with notable preservation of pulp vitality, an important distinction to be made in considering probable clinic applications (Neves & Sharpe, 2018). In a later study, impressive extents of reactionary dentin remineralization emulating thickness levels seen in healthy non-damaged molars, were observed at carious lesion sites bereft of pulp exposure in mouse molars treated with Tideglusib, compared to the minimal mineralization recorded in control molars treated with collagen sponges devoid of the GSK3 antagonist (Neves et al., 2017). Evidence from these studies support the intrinsic ability of small-molecule drugs like Tideglusib to infiltrate regions of damaged dentin and act on the naturally restorative capacities of odontoblasts and pulp cells for advanced healing in large dental lesions exhibiting dentin and/or pulp exposure. The Wang laboratory showed that another small molecule GSK3-inhibitor, CHIR99021, prompted upregulation of Wnt/β-catenin signaling to induce vasculogenic differentiation of DSPCs in immunodeficient mice, as evidenced by a marked increase in the expression of endothelial components VEGFR2, VE-Cadherin, CD31, and Tie-2 (Zhang et al., 2016). Mesenchymal DPSCs were employed as fundamental precursor cells in the *de novo* generation of primordial endothelium imperative for initial proliferation of new vascular networks. This type of vasculogenesis using DPSCs, or even bone marrow mesenchymal stem cells (BMSCs) (Xu et al., 2008), has elucidated a plausible method for the secondary neovascularization of dental tissues following events of expansive trauma or defect. In this same study, it was seen that use of the small molecule inhibitor JW67, specifically in the repression of Wnt/β-catenin signaling, enhanced odontoblast and osteoblast differentiation of DPSCs. These findings raised interest around the implications of Wnt/β-catenin acting as an amenable pathway for the osteogenic and/or vasculogenic differentiation of mesenchymal stem cells, deriving potential therapeutic benefit as a mechanism for facilitating craniofacial or dentoalveolar regeneration. In regard to the misconfiguration and underdevelopment of the palatal bones seen in cleft palate, some small molecular Wnt agonists have been indicated in the disruption of congenital clefting during embryonic formation of the palate. Examples of such small molecules include the *DKK1* inhibitors WAY-262611 and IIIc3a which effectively preserved the cleft phenotype in *Pax9* ^−/−^ *Dkk1* ^*f/+*^; *Wnt1Cre* mouse embryos by encouraging complete and partial fusion between the primary and secondary palates. Controlled intravenous delivery of these two Wnt agonist small molecules into pregnant *Pax9* ^*+/−*^ mice amends a majority of cleft palate defects in mutant offspring. Treatment with WAY-262611 from embryonic days 10.5–13.5 rescued the cleft phenotype, though to a lesser extent than observed during regimented dosing from embryonic days 10.5–14.5. Of those mutant progenies studied, ∼60% (11/18) displayed complete fusion between the primary and secondary palates while the other 7/18 showed small residual defects at the zone of fusion for the primary and secondary palates near the 3rd palatal rugae. Administration of IIIc3ac to pregnant *Pax9* ^*+/−*^ mice from embryonic days 10.5–14.5 showed successful closure of the secondary palate defects in 80% (12/15) of mutant embryos, of which six embryos retained residual fusion defects. Although the corrective properties of both *DKK1* inhibtors were highlighted in this study, it was also observed that Wnt agonist therapies using WAY-262611 did not prevent defects in tooth organs and other structures affected in modified mouse embryos (Jia et al., 2017). Primary outcomes for in vivo trials are outlined in Table 2.

**TABLE 2:** Small molecular agents examined *in vivo*.

Molecule	Classification	Molecular Weight	Working Concentrations	Bone Defect/Surgical Model	Delivery Methods	Primary Osteogenic Effect(s)	References
SB431542	Benzamides	384.4 g/mol	0.1 μM, 1.0 μM	Guangxi Bama minipigs with maxillary bone defects (12 mm x 5 mm - Diameter x Depth) produced using a trephine	Minipig GMSC grafts treated with SB431542 were implanted into the maxillary defect	Downregulates TGF-β signaling; prevents Smad3 gene phosphorylation; induces activation of BMP signaling in minipigs	Shi et al. (2019)
.	.	.	26 mM	CD-1 immunodeficient mice with parietal bone defects (2 mm in diameter) produced using a trephine	Collagen sponges treated with SB431542 were implanted into the parietal bone defect	Enhanced parietal bone regeneration within the calvarium; reduced Smad2 and Smad3 expression	Senarath-Yapa et al. (2016)
Phenamil	Amiloride derivative	305.7 g/mol	300 μM	Sprague Dawley rats with critical-sized mandibular defects (5 x 5 x 2.5 mm3) produced using a drill burr	Appropriately-sized scaffolds with phenamil were placed onto the mandibular defect with a resorbable suture, followed by skin closure with nonresorbable suture	Inhibition of Smurf1 in rats	Fan et al. (2017b)
Resveratrol	Stilbenes	228.2 g/mol	1.0 mg/ml	Sprague Dawley rats with critical-sized mandibular defects (circular, full-thickness 4-mm) produced using a trephine	RSV-SrRn scaffolds were impalnted into the mandibular defect before being closed	Higher bone volume to total volume (BV/TV) ratios; peripheral and central bone regeneration inrats with large mandibular defects	Zhang et al. (2020)
Strontium ranelate	Tetracarboxylic acids	513.5 g/mol	1.0 mg/ml	Sprague Dawley rats with critical-sized mandibular defects (circular, full-thickness 4-mm) produced using a trephine	RSV-SrRn scaffolds were implanted into the mandibular defect before closure of defect	Higher bone volume to total volume (BV/TV) ratios; peripheral and central bone regeneration in rats with large mandibular defects	Zhang et al. (2020)
Simvastatin	Statins	418.6 g/mol	Not reported	Wistar rats with exposed tooth socket after extraction of mandibular right central incisor	PLGA scaffolds with simvastatin were implanted in the tooth sockets before suturing of the gingiva	Induced BMP2 gene expression; anti-resorption elements to impede osteoclast proliferation in rats	Wu et al. (2008)
Dimethyloxalylglycine	Glycine derivative	175.1 g/mol	1% w/w of PLGA	Wistar rats with mandibular buccal bone defects (5 × 4 × 1 mm3) produced using a drill burr	Fibrous membranes containing DMOG were implanted into mandibular defect, then the defect was closed	Stimulation of PDLSC osteogenic differentiation; modulation of deleterious inflammatory responses in rats	Shang et al. (2021)
Tideglusib	Naphthalenes	334.4 g/mol	50 nM	Axin2 and GPR177(Wntless) mice with exposed dentin or pulp on maxillary first molars produced using a drill burr	Exposed dentin on maxillary first molars were capped with tideglusib	Tertiary dentin formation; Wnt/β-catenin pathways upregulation in odontoblastic pulp cells of mouse molars	Neves & Sharpe (2018); Neves et al. (2017)
CHIR99021	Aminopyrimidines	465.3 g/mol	Different concentrations between 0 μM and 10 μM	Severe combined immunodeficient (SCID) mice	Tooth slices/scaffolds containing DPSCs, treated with CHIR99021, were transplanted into the subcutaneous space of the dorsum of SCID mice	Wnt/β-catenin signaling upregulation; increased VEGFR2, VE-Cadherin, CD31, Tie-2 for vasculogenic differentiation of DSPCs in mice	Zhang et al. (2016)
JW67	-	394.4 g/mol	Different concentrations between 0 μM and 10 μM	Severe combined immunodeficient (SCID) mice	Tooth slices/scaffolds containing DPSCs, treated with JW67, were transplanted into the subcutaneous space of the dorsum of SCID mice	Enhanced odontoblast and osteoblast differentiation of DPSCs in mice	Zhang et al. (2016)
HA-1077 (Fasudil)	Isoquinoline derivative	291.4 g/mol	0.5 μM	Sprague Dawley rats with parietal bone defects (circular, 5mm in diameter) produced using a trephine	Atelocollagen sponges containing HA-1077 were implanted into the parietal bone defect, then the flap was sutured.	Enhanced parietal bone regeneration within the calvarium	Nakata et al. (2020)
Y-27632	N-arylamides	247.3 g/mol	0.5 μM, 5 μM	Sprague Dawley rats with parietal bone defects (circular, 5 mm in diameter) produced using a trephine	Atelocollagen sponges containing Y-27632 were implanted into the parietal bone defect, then the flap was sutured.	Enhanced parietal bone regeneration within the calvarium; upregulated VEGF-A mRNA expression	Nakata et al. (2020)
Dexamethasone	Hydroxysteroids	392.5 g/mol	2.18 μg/mg of chi-MSNs	Sprague Dawley rats with skull defects (5 mm in diameter), tools used not reported	Gelfoam implants containing DEX-embedded silica nanoparicles were transplanted to the defect site	Higher bone volume to total volume (BV/TV) ratios; enhanced skull bone regeneration	Sun et al. (2019)
Hydroxycholesterol mixture*	Oxysterols	see footnote	5 μM	Sprague Dawley rats with alveolar clefts from zygomatic arch to the ipsilateral maxillary incisor (7 × 4 × 3 mm3), produced using a hand-held drill and silicone molds for accuracy	Collagen sponges containing hydroxycholesterols were implanted into the alveolar defect, then the gingival flap was sutured.	Enhanced alveolar cleft regeneration; absence of adverse infiltration by inflammatory cells	Bakshi et al. (2019)
WAY-262611	Naphthalenes	318.4 g/mol	12.5 mg/kg, 25 mg/kg per mouse body weight	Pax9 -/- Dkk1 f/+ ;Wnt1Cre compound mutant mice embryos (generated through serial matings) displaying cleft palates	Doses of WAY-262611 were injected daily into the tail veins of pregnant Pax9 +/- mice	Increased Wnt signaling; enhanced cell proliferation; palatal shelf outgrowth and fusion; upregulation in Col1a1 expression in postnatal palatal mesenchyme	Jia et al. (2017)
IIIc3a	-	570.1 g/mol	12.5 mg/kg, 25 mg/kg per mouse body weight	Pax9 -/- Dkk1 f/+ ;Wnt1Cre compound mutant mice embryos (generated through serial matings) displaying cleft palates	Doses of WAY-262611 were injected daily into the tail veins of pregnant Pax9 +/- mice	Increased Wnt signaling; enhanced cell proliferation; palatal shelf outgrowth and fusion; upregulation in Col1a1 expression in postnatal palatal mesenchyme	Jia et al. (2017)

*20(S)-hydroxycholesterol (402.7 g/mol) and 22(R)-hydroxycholesterol (402.7 g/mol).

## Concluding remarks and future directions

**FIGURE 4:**
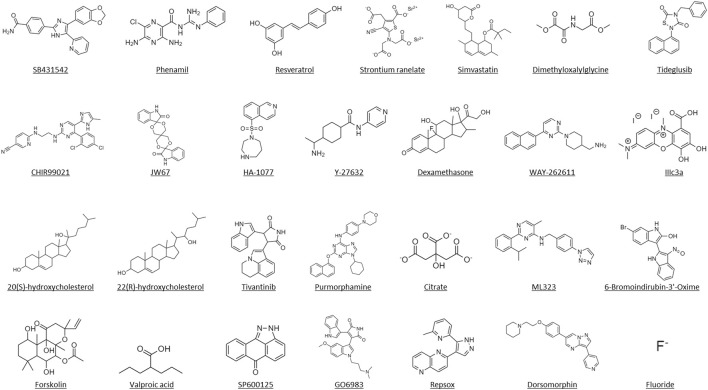
Molecular structures of the identified small molecules that have been investigated for *in vitro* and *in vivo* craniofacial and dentoalveolar tissue engineering.

Craniofacial and dentoalveolar defects are major bone diseases affecting millions of individuals worldwide, yet the efficacy and safety of current treatment methods are limited and present significant complications that prevent their unmarred integration into patient care. Small molecule mediated-bone regeneration has been proposed as a promising strategy due to the inherent characteristics of small molecules that can minimize or even bypass the limitations observed in both protein-based therapeutics and conventional means of obturation such as high manufacturing cost, protein instability and degradation, risk of contamination, and unwanted immune response (Lu and Atala, 2014). We provide a review for the prospects of small molecules mediated craniofacial and dentoalveolar bone regenerative engineering. We also review the preclinical study of small molecules associated with craniofacial and dentoalveolar bone regeneration. In fact, over the past decade, a number of small molecules with bone regenerative potential have been reported in the literature, these small molecule compounds include known pharmaceutical bioactive compounds and unknown novel small molecule compounds (Figure 4). Although many of these efforts have focused on identifying small molecules that augment BMP-2 mediated osteoblast function (Fan et al., 2015; Fan et al., 2017a; Moon et al., 2017; Qi et al., 2017) there has been less emphasis on identifying small molecules alone that are able to stimulate primitive and undifferentiated cells to develop into the osteogenic cell lineage with the capacity to form new bone. Therefore, it is important to identify small molecules with potent inherent osteoinductivity without the need of exogenous BMP recombinant protein supplementation.

Drug repositioning is the application of approved small molecule drugs for new indications (Jourdan et al., 2020). An advantage of drug repositioning over traditional drug development is that since the repositioned drugs have already passed a significant number of toxicity and other tests, their safety is known and the risk of failure for reasons of adverse toxicology are significantly reduced (O’Neill et al., 2019). Herein, a new drug repositioning method should be performed that is able to identify approved small molecule-based drug(s) with potent osteoinductivity inherent without the need of exogenous BMP recombinant protein supplementation. In addition, artificial intelligence (AI) tools are starting to enable pharmaceutical researchers to search chemistry from expansive small molecule databases for developing drugs at a much faster rate than ever before (Sharma and Sharma, 2018).

It is also worth mentioning that stem cells hold great promise for regenerative therapies for a wide spectrum of diseases or injuries by virtue of their ability to regenerate tissues and contribute to their homeostasis (Bacakova et al., 2018). Interestingly, deciduous teeth contain a rich supply of stem cells in their dental pulp. These cells called stem cells from human exfoliated deciduous teeth (SHEDs) are highly proliferative, clonogenic and capable of differentiating into a variety of cell types including osteoblasts and odontoblasts (Miura et al., 2003; Yang et al., 2019). It is believed that the great potential of SHEDs for repairing and regenerating bone tissue would certainly bring basic science concept closer to clinical studies. In addition, it is possible to investigate the potential of osteogenic small molecule in conjunction with SHEDs for engineering bone tissue for dental as well as orthopaedic applications. An effective delivery approach for small molecules is another objective of small molecule-based bone regenerative engineering. Given that small molecule compounds can be formulated into numerous forms of biomaterials (Laurencin et al., 2014), thus it is vital to develop an engineered scaffold delivery system that provides adequate doses of the small molecule and/or acts as a structural support for infiltrating cells (Lo, 2022).

## Abbreviations

GSK3, Glycogen synthase kinase-3; Dspp, Dentin sialophosphoprotein; OCN, Osteocalcin; DPSC, Dental pulp stem cell; OPN, Osteopontin; SCAP, Stem cells from apical papilla; PHD, Prolyl hydroxylases; GMSC, Gingival mesenchymal stem cells; DMOG, Dimethyloxalylglycine; nSi, Nanosilicate; BSP, Bone sialo-protein; PLGA, Polylactic acid/polyglycolic acid; RSV, Resveratrol; PDLSC, Periodontal ligament stem cells; SrRn, Strontium ranelate; MSC, Mesenchymal stem cell; Trib3, Tribbles homolog 3; CPC, Calcium phosphate cement; SV, Simvastatin; BMP-2, Bone morphogenetic protein-2; PCL/TCP, Polycaprolactone/β-tricalcium phosphate; hiPSCs, Human-induced pluripotent stem cells; BMSC, Bone marrow mesenchymal stem cells; Smurf1, Smad ubiquitin regulatory factor 1; PPARγ, Peroxisome proliferator activated receptor gamma; SHED, Stem cells from human exfoliated deciduous teeth; BIO, 6-Bromoindirubin-3′-Oxime.
